# Differentiated Empowering Leadership and Interpersonal Counterproductive Work Behaviors: A Chained Mediation Model

**DOI:** 10.3390/bs14090760

**Published:** 2024-08-28

**Authors:** Yuanzhao Song, Haining Zhou, Myeong-Cheol Choi

**Affiliations:** 1 Department of Business, Gachon University, Seongnam 13120, Republic of Korea; soongyuen@foxmail.com; 2 School of Journalism and Communications, Shandong Normal University, Jinan 250014, China

**Keywords:** differentiated empowering leadership, trust in leaders, defensive silence, interpersonal counterproductive work behaviors

## Abstract

Through an empirical analysis of paired sample data from 308 employees in China, this study examines the chain-mediated effects of trust in leaders and defensive silence on the relationship between differentiated empowering leadership and interpersonal counterproductive work behaviors. The study finds that differentiated empowering leadership does not directly influence employees’ interpersonal counterproductive work behaviors. Additionally, it finds that trust in leaders and defensive silence each serve as mediators in the relationship between differentiated empowering leadership and interpersonal counterproductive work behaviors, forming a chained mediation effect. This study is the first empirical research to explore the impact mechanism of differentiated empowering leadership using a chained mediation model. The findings contribute to a deeper understanding of how and why differentiated empowering leadership affects employees’ attitudes, such as trust in leaders, and behaviors, such as interpersonal counterproductive work behaviors.

## 1. Introduction

The study of differentiated leadership behavior first appeared in the literature on leader–member exchange (LMX), confirming that leaders treat their followers differently [1]. It has since been expanded to research transformational, empowering, and servant leaderships [2,3,4]. This study focuses on the differentiation of empowering leadership for two reasons. First, with the advent of the era of organizational flattening, it is becoming increasingly difficult for autocratic leadership to adapt to and dynamically match the environment it faces. Empowering leadership has become a widely used leadership style in organizational management. This leadership style has been shown to help unleash employees’ subjective initiatives and improve their self-efficacy [3]. Notably, due to the limited energy and resources of leaders, they grant different levels of power and autonomy to different subordinates. Additionally, influenced by circular and hierarchical patterns in Confucian culture, differentiated empowering leadership behavior is particularly common in East Asian cultural circles. Second, scholars have recently found that leader empowerment is not always beneficial. The degree and scope of empowerment can have different effects on employees’ attitudes and behaviors. For example, insufficient empowerment reduces employees’ job autonomy, leads to work burnout, weakens employees’ harmonious passion, and affects job performance and organizational citizenship behavior [5]. Conversely, excessive empowerment increases uncertainty in the work environment, making it difficult for employees to form clear role identities, leading to emotional exhaustion and ultimately reducing employees’ self-efficacy and job satisfaction [6]. Therefore, a more comprehensive view of the impact of empowering leadership is needed. This study utilizes social comparison theory to explain the effects of differentiated empowerment (a team-level concept) on employee attitudes and behaviors. This approach will help explore the boundary conditions for the effectiveness of empowering leadership styles.

Research indicates that leaders’ positive attitudes towards subordinates improve employees’ attitudes towards work, leadership, and the organization. Conversely, poor leadership styles are one of the main reasons for employee turnover and deviant behavior [7]. Hostile relationships between leaders and subordinates can lead to a loss of employee identification and psychological safety towards the leader [8]. This lack of trust can diminish their organizational citizenship behavior [9]. Lower levels of trust may even manifest as emotional exhaustion, employee silence, counterproductive behaviors, and other deviant behaviors [10].

Counterproductive work behavior (CWB) refers to deliberate actions by employees that go against the legitimate interests of the organization or its members [11]. CWB is categorized into organizational counterproductive work behavior (OCWB) and interpersonal counterproductive work behavior (ICWB) [12]. OCWB includes behaviors that harm the organization, such as withdrawal, sabotage, tardiness, and leaving work early. ICWB involves behaviors that negatively affect other individuals within the organization, such as gossiping, criticizing others’ opinions, and creating discord among colleagues. Research indicates that work environment variables, such as psychological contract breach, interpersonal conflict, organizational justice, and organizational constraints, have a more significant impact on ICWB than on OCWB [13,14]. The concept of ICWB is more relevant to this study, which focuses on the relationship between differentiated empowering leadership and employees’ attitudes and behaviors toward colleagues or leaders. Thus, we select ICWB as the outcome variable for this research. This study, based on social exchange theory, examines the association between differentiated empowering leadership-induced employee distrust and negative behaviors and ICWB.

This study aims to address two primary research gaps. The first gap concerns differentiated empowering leadership. Current research on leadership empowerment primarily emphasizes its positive impacts on emotions and motivation, while potential negative outcomes are relatively understudied. Only a few researchers have investigated this direction, demonstrating that differentiated empowering leadership can provoke negative emotions, trigger intra-team relational conflicts, and reduce employees’ helping behaviors [3,4,15]. This indicates the need for further exploration in this area. The second gap involves the mechanisms linking antecedent variables to ICWB. Existing studies often explain the antecedents of employee counterproductive behaviors from moral and stressor perspectives. However, there is a scarcity of research explaining the mechanisms between antecedent variables and ICWB from the perspective of social exchange. For instance, Cohen and Diamant [16] proposed that job burnout and organizational politics lead to higher levels of interpersonal counterproductive behaviors from an interpersonal stressor perspective. Zhao et al. [17] discussed the mediating role of moral disengagement between unethical tasks and retaliatory behaviors. Bellora-Bienengräber et al. [18] explored how management control systems convey moral values to curb negative employee behaviors from a moral perspective. In this case, we emphasize the impact of trust in leaders and defensive silence as antecedents influencing ICWB, thereby enriching the body of research on ICWB.

In summary, this study utilizes social exchange theory and social comparison theory to design a chained mediation model. The study sets out the following objectives:

1. To investigate the impact of differentiated empowering leadership on ICWB.
2. To explore the influence of differentiated empowering leadership on trust in leaders and defensive silence.
3. To examine whether differentiated empowering leadership affects ICWB through the mediating effects of trust in leaders and defensive silence.

## 2. Theory and Hypotheses

### 2.1. Differentiated Empowering Leadership and Interpersonal Counterproductive Work Behaviors

Differentiated Empowering Leadership refers to team leaders’ differential treatment of team members, including the unequal distribution of power, organizational care, and organizational support [19]. Scholars have demonstrated that leaders’ differential empowerment behaviors have a positive impact on employees’ negative emotions, such as depression [15]. Differences in job resources, such as salary and authority, can trigger competition and comparison among employees, affecting their perceived justice, psychological pressure, and counterproductive work behavior [4]. Research on LMX has also shown that a higher degree of differentiation in empowerment results in the division of teams into two groups: an “in-group” that receives more empowerment and an “out-group” that receives less empowerment [4]. In-group members receive more leader care, job autonomy, and other job-related resources, exacerbating the unfair distribution of workplace benefits, such as promotions and salaries. When employees engage in upward comparisons with their in-group colleagues, who receive more empowerment in terms of job-related resources, employees who perceive themselves as being treated unfairly are more likely to experience negative emotions such as jealousy, anger, and humiliation [20,21].

Mbah and Ikemefuna [7] emphasized that poor leadership styles are one of the main reasons that employees engage in deviant behaviors. According to social comparison theory, individuals tend to engage in upward social comparisons, and when they perceive themselves as being at a disadvantage, it can lead to feelings of frustration. Consequently, individuals often take action to alleviate these negative emotions. In such situations, employees may engage in ICWB, sabotage others’ work performance and interpersonal relationships, and intentionally harm the legitimate interests of the organization. ICWB as a form of deliberate deviance from organizational norms, has a detrimental impact on the well-being of other members within the organization. Dalal [12] define ICWB as “a range of harmful behaviors that intentionally violate organizational norms and may harm other members within the organization, such as colleagues and leaders”. Typical ICWB behaviors include badmouthing colleagues behind their backs, publicly belittling their opinions, and deceit [22]. These actions often stem from employees’ perceptions of unfair differentiated empowerment, negatively affecting extra-role helping behaviors such as team collaboration and knowledge sharing [23]. Moreover, from a practical standpoint, ICWB directly impacts employee work efficiency, task performance, and even team performance [24,25]. The harmful consequences and substantial costs associated with ICWB span all stages of human resource management.

Previous research indicates that ICWB is inevitably influenced by the quality of interpersonal relationships [24,26]. Low-quality interpersonal relationships are seen as exploitative and positively correlated with dysfunctional workplace behaviors [27]. This relationship also supports equity theory. When employees perceive distributive injustice, high levels of job burnout increase employee aggression and emotionality, leading to higher levels of ICWB [16]. There is also a significant interaction between psychological contract breach and ICWB [16]. Additionally, ICWB can be predicted by interpersonal conflict [24]. Incompetent leadership exacerbates interpersonal conflict, reduces job satisfaction, increases turnover intentions, and heightens the likelihood of engaging in counterproductive work behaviors [28]. Based on this, we hypothesize that differentiated treatment by leaders will intensify the unfair distribution of job resources such as promotions and salaries, and disadvantaged employees will engage in ICWB as a form of retaliation. Therefore, this study proposes the following hypothesis:

**Hypothesis** **1.** *Differentiated empowering leadership positively influences employees’ interpersonal counterproductive work behaviors.*

### 2.2. The Mediating Role of Trust in Leaders

Trust binds leaders and subordinates closely together, with the level of trust subordinates have in their leaders being a crucial factor in determining the effectiveness of leadership [29]. Similarly, trust in leaders is vital for encouraging employees to engage in extra-role behaviors beyond their legal and contractual obligations [30]. Previous literature suggests that empowering leadership fosters greater trust in leaders among subordinates [31]. This is because leaders’ attention to subordinates, allocation of autonomy, and other work resources enhance perceptions of justice and trustworthiness towards the leader [32]. This represents a positive exchange relationship. Hence, empowering leadership is generally positively correlated with trust in the leader.

Unlike previous studies, we focus on differentiated empowerment. Rousseau et al. [33] emphasized that trust as a psychological state depends on specific social exchange relationships. In other words, subordinates’ trust in their leader depends on the leader’s behavior [34]. When leaders fail to formulate policies or distribute resources based on principles of internal consistency, subordinates’ trust in them can be compromised [29]. Muafi et al. [35] demonstrated that perceived fairness of interpersonal treatment is crucial in explaining the relationship between leaders’ empowerment and subordinates’ trust in their leaders. Therefore, we hypothesize that differentiated empowering leadership negatively impacts subordinates’ trust in their leaders.

Additionally, leaders’ empowerment reflects their evaluation of their employees [36]. Therefore, when employees perceive lower levels of autonomy being granted to them, they may interpret this as a lack of leaders’ attention or trust in them [37]. Subordinates develop trust in leaders because they perceive trust, care, and respect from them. In other words, perceived trust, care, and respect from leaders all play a more proactive role in the leader–subordinate relationship [38]. When subordinates perceive their leaders as insincere and untrustworthy, they reduce their organizational citizenship behavior [9]. Lower levels of trust can even lead to counterproductive work behaviors [10]. Krischer et al. [39] also found that when employees perceive a lack of trust in an organization, they attempt to compensate for resources to some extent through counterproductive work behaviors such as passive resistance and malicious competition. Research on LMX has also shown that differentiated treatment disrupts perceived justice, and employees’ psychological perceptions ultimately influence their behavioral responses [40]. Based on this, we argue that trust in leaders is a guiding mechanism for employee attitudes and behaviors. Differentiated leadership behavior logically influences employees’ ICWB by undermining trust in leaders. Therefore, we propose the following hypothesis:

**Hypothesis** **2.** *Trust in leaders mediates the relationship between differentiated empowering leadership and interpersonal counterproductive work behaviors.*

### 2.3. The Mediating Role of Defensive Silence

Employee silence does not only refer to employees lacking speech. Instead, employee silence refers to the intentional concealment of thoughts, information, or opinions regarding potential improvements within an organization. Dyne et al. [41] categorize employee silence into three types: prosocial silence, acquiescent silence, and defensive silence. Prosocial silence is considered a form of organizational citizenship behavior where employees withhold their views based on altruistic or cooperative motives [42]. Acquiescent silence is a relatively passive form of compliance where employees believe they lack the ability to change the organization’s current state and therefore conceal their views. Defensive silence is a self-protective form, where employees withhold relevant thoughts, information, or opinions out of fear [41]. It is negatively correlated with psychological safety [43]. When employees perceive that sharing information may be unsafe, uncertain, or threatening, they consciously choose to remain silent to avoid potential interpersonal conflicts that could arise from expressing their opinions [44,45,46]. These conflicts may occur among colleagues but are more frequently seen between leaders and subordinates [47]. In this study, we specifically focus on defensive silence as a proactive form of silence.

Leadership behavior is an important organizational environmental factor in predicting employee silence [48]. According to social exchange theory, relationships between individuals are based on the principle of reciprocity. This means that if leaders solicit employee opinions, encourage empowerment, and involve employees in decision-making, employees will also be willing to provide information and suggestions for organizational development. Even if offering suggestions may jeopardize their own interests and interpersonal relationships, employees are willing to take such risks because of the psychological rewards in social exchange [48,49,50].

In contrast, destructive leadership styles can trigger intense negative emotions and lead to coping strategies such as reduced work effort or reduced communication with leaders [45]. Destructive leadership styles, such as narcissistic, autocratic, exploitative, and abusive supervision, have been identified as potential antecedents of defensive silence [8,51]. Scholars have emphasized that when employees seek to avoid conflict and protect themselves, they tend to adopt avoidance or passive coping strategies [52]. For example, they may reduce interpersonal interactions to avoid potential disputes from external environments [53]. Alternatively, they may engage in feedback avoidance behaviors and actively avoid or resist leadership [54]. Furthermore, Jung and Yoon [55] argued that employee silence can lead to various forms of deviant behavior, including disobedience, insults, criticism of colleagues, rumors, and lies, among other ICWB. This is because employees who remain silent out of fear exhibit a stronger intention to leave, thereby increasing the likelihood of deliberately engaging in counterproductive behaviors [56]. Thus, we propose the following hypothesis:

**Hypothesis** **3.** *Defensive silence mediates the relationship between differentiated empowering leadership and interpersonal counterproductive work behaviors.*

### 2.4. The Chained Mediation Role of Trust in Leaders and Defensive Silence

According to the social exchange theory, when leaders solicit employee opinions, encourage empowerment, and involve them in decision-making, employees perceive a positive social exchange relationship. This can make employees feel trusted and valued [57]. Subordinates develop trust in leaders because they perceive trust from their leaders [38]. Therefore, to reciprocate the trust and value shown by leaders, employees, driven by the reciprocity principle in social exchange, reduce their defensive silence behaviors of withholding their viewpoints [49]. This has positive effects on motivating employees’ attitudes and behaviors, including job satisfaction, work motivation, organizational commitment, and organizational citizenship behaviors [58,59]. Relevant studies have also confirmed the close relationship between trust in leaders and defensive silence [50]. Based on this, we propose the following hypothesis:

**Hypothesis** **4.** *Trust in leaders and defensive silence mediate the relationship between differentiated empowering leadership and interpersonal counterproductive work behaviors in a chained mediation model.*

**Figure 1:**
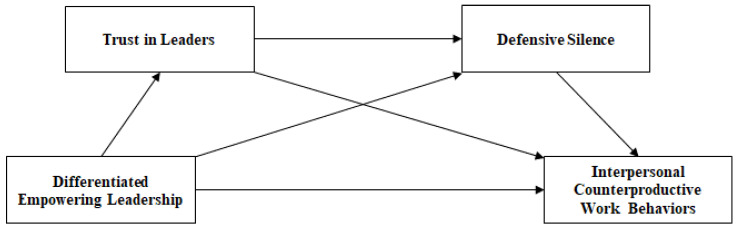
Research model.

The research model of this study is illustrated in Figure 1.

## 3. Methods

### 3.1. Participants and Procedure

Participants were selected from companies and organizations in the Shandong and Jiangsu provinces of China. The industries involved in this study include the internet, real estate, and manufacturing industries, among others. We adopted a paired-sample collection method to reduce potential common method biases. The variables of differentiated empowering leadership, trust in leaders, and defensive silence were evaluated by the respondents themselves. ICWB was assessed by colleagues familiar with the same team or department. Survey participants also provided demographic information such as gender, age, education level, and work experience. Before distributing the questionnaires, the researchers provided detailed instructions to participants. The participants were assured that the survey was only for academic research purposes and we would not divulge any commercial secrets or privacy. They were also informed that there were no right or wrong answers and that their responses would be kept strictly confidential. After collecting the questionnaires, the participants’ self-assessment questionnaires were paired with colleagues’ evaluation questionnaires to ensure matching and confidentiality.

The survey was conducted between March and April 2024. A total of 449 questionnaires were distributed to 67 teams. Of these, 5 teams had missing paired responses, failing to reflect the actual team situations, and thus were excluded. Additionally, one team had fewer than 3 members, leading to insufficient statistical power of the sample size, so it was also excluded. Ultimately, 402 questionnaires were collected from 61 teams. After excluding questionnaires with missing data and excessively identical responses and patterns in the answers, 308 valid questionnaires were selected, resulting in a response rate of 89.5% and an effective questionnaire rate of 76.6%. Among the 308 valid questionnaires, 55.8% were from male participants, and 44.2% were from female participants. The majority of respondents were under 30 years of age, accounting for 46.7% of the total sample. The next largest age group was 31–40, accounting for 37.3% of the total sample. Employees aged 41–50 and above 50 accounted for only 9.7% and 6.2% of the sample, respectively. In terms of education level, most respondents had received a good education: 39.3% had a bachelor’s degree, and 24.4% had a graduate or higher degree. Additionally, 28.9% had an associate’s degree and only 7.5% of the total sample had a high school education or lower. In terms of work experience, most respondents had less than five years of experience, accounting for 43.2% of the total sample. The second-largest group comprised employees with 6–10 years of work experience, accounting for 23.1% of the total sample. Employees with 11–15 years, 16–20 years, and more than 20 years of work experience were relatively few, accounting for 11.4%, 10.7%, and 11.7% of the total sample, respectively.

### 3.2. Measures

#### 3.2.1. Differentiated Empowering Leadership

Differentiated empowering leadership is a team-level concept. We followed the recommendation of Harriso and Klein [60] and used the within-group coefficient of variation (CV) developed by Chan [61] as the measurement indicator for differentiated empowering leadership. The calculation method was the standard deviation of team members’ scores on empowering leadership divided by the mean. The measurement of differentiated empowering leadership utilized the 12-item scale developed by Ahearne et al. [62]. The items included statements such as “My leader frequently involves me in decision-making” and “If a decision could affect me, my leader asks for my opinion beforehand” (α = 0.941).

#### 3.2.2. Trust in Leaders

The 5-item scale measuring trust in leaders was adapted from Podsakoff et al. [63]. The items included statements such as “I have confidence that my leader will treat me fairly” and “I completely trust the integrity of my leader” (α = 0.910).

#### 3.2.3. Defensive Silence

The 5-item scale measuring defensive silence was adapted from Dyne et al. [41]. The items included statements such as “I withhold relevant information out of fear” and “I avoid expressing ideas that might change the status quo for self-protection” (α = 0.883).

#### 3.2.4. Interpersonal Counterproductive Work Behaviors

The 6-item scale measuring counterproductive work behavior was adapted from Dalal et al. [64]. The scale was assessed through peer ratings and included items such as “They frequently criticize the opinions or suggestions of leaders/colleagues” and “They frequently avoid interacting with leaders/colleagues” (α = 0.920).

## 4. Results

### 4.1. Common Method Variance Assessing

We conducted Harman’s single-factor test to explore the potential for common method bias. After conducting a factor analysis with rotation on all items in the questionnaire, if only one factor emerged or if the first factor explained the majority of the variance, it would indicate a significant common method bias [65]. We employed principal component analysis and obtained four factors with eigenvalues greater than 1. However, the first factor explained 26.283% of the total variance, which is below the critical threshold of 50%. This indicates that the study did not encounter a situation in which a single factor explained most of the variance. Therefore, we conclude that the common method bias issue is not severe in the sample data collected for this study.

### 4.2. Reliability and Validity

First, the reliability analysis revealed that all variables in this study had Cronbach’s α coefficients exceeding 0.800, indicating high internal consistency for each variable. The CR values for differentiated empowering leadership, trust in leaders, defensive silence, and ICWB were 0.942, 0.913, 0.882, and 0.921, respectively, all surpassing the critical threshold of 0.800. The AVE values were 0.576, 0.677, 0.604, and 0.661, all of which exceeded the critical threshold of 0.500. This indicates good convergent validity for all the variables in this study.

Second, we conducted confirmatory factor analysis to assess the discriminant validity of the measurement scales. The relative chi-square, CFI, IFI, TLI, and RMSEA were used as indicators of model fit. The four-factor model (*χ*^2^/*df* = 2.905, CFI = 0.894, IFI = 0.895, TLI = 0.884, RMSEA = 0.079) demonstrated a significantly better fit than other factor models, indicating good discriminant validity of the measurement tool. Additionally, the single-factor model (*χ*^2^*/df* = 10.310, CFI = 0.474, IFI = 0.476, TLI = 0.432, RMSEA = 0.174) exhibited the worst fit, further indicating the absence of significant common method bias in the study data.

### 4.3. Correlation Analysis

Table 1 summarizes the means, standard deviations, and correlation coefficients among demographic variables, differentiated empowering leadership, trust in leaders, defensive silence, and ICWB. In terms of the relationship between the demographic and main variables, employee age is significantly correlated with ICWB (r = −0.259, *p* < 0.01). Education level is significantly correlated with differentiated empowering leadership (r = 0.141, *p* < 0.05) and ICWB (r = −0.149, *p* < 0.01). Work tenure is significantly correlated with ICWB (r = −0.258, *p* < 0.01). Therefore, the influence of demographic variables must be considered in subsequent regression analyses.

Among the four main variables, differentiated empowering leadership is significantly negatively correlated with trust in leaders (r = −0.324, *p* < 0.01) and positively correlated with defensive silence (r = 0.291, *p* < 0.01). However, this is not significantly correlated with ICWB. Trust in leaders is significantly negatively correlated with defensive silence (r = −0.362, *p* < 0.01) and ICWB (r = −0.229, *p* < 0.01). Defensive silence is significantly and positively correlated with ICWB (r = 0.391, *p* < 0.01).

### 4.4. Tests of Hypotheses

**Figure 2:**
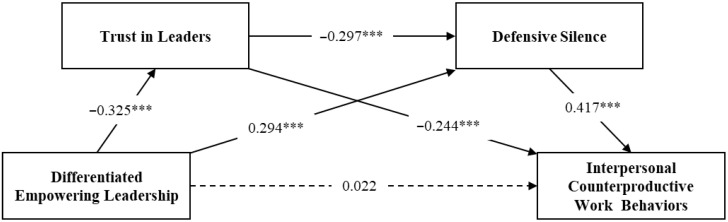
Path analysis diagram. *** *p* < 0.001.

The results of the collinearity analysis show that the maximum VIF value for the main variables is 1.238. This indicates that there are no serious collinearity issues, and the survey data’s analysis results are reliable. Based on this, a regression analysis is conducted (see Table 2). Figure 2 illustrates the specific path coefficients of the mediation model. In the total effect model, the impact of differentiated empowering leadership on ICWB is not significant (β = 0.022, *p* > 0.05). However, differentiated empowering leadership had a significant effect on trust in leaders (β = −0.325, *p* < 0.001) and defensive silence (β = 0.294, *p* < 0.001). The impact of trust in leaders on defensive silence is also significant (β = −0.297, *p* < 0.001). Additionally, trust in leaders (β = −0.244, *p* < 0.001) and defensive silence (β = 0.417, *p* < 0.001) had significant effects on ICWB.

Further, the PROCESS (Model 6) bootstrap test was conducted, with 5000 resamples and a 95% confidence interval [66]. The results are presented in Table 3. First, we performed a linear regression with 5000 bootstrap samples to examine the direct effect of the independent variable on the dependent variable. The results show that the effect of differentiated empowering leadership on ICWB is not significant (95% CI = −0.960 to 0.656), thereby failing to support Hypothesis 1.

Second, Hypotheses 2 and 3 test the mediating effects of trust in leaders and defensive silence. The results indicate that differentiated empowering leadership has a significant indirect effect on ICWB through trust in leaders (β = 0.302, 95% CI = 0.034–0.614), thus supporting Hypothesis 2. Similarly, differentiated empowering leadership has a significant indirect effect on ICWB through defensive silence (β = 0.530, 95% CI = 0.202 to 0.912), supporting Hypothesis 3. Hypothesis 4 tests the chained mediation effect of trust in leaders and defensive silence. The results show that differentiated empowering leadership has a significant indirect effect on employees’ ICWB through the chained mediation of trust in leaders and defensive silence (β = 0.259, 95% CI = 0.118 to 0.435). Therefore, Hypothesis 4 is supported.

Finally, by comparing the data in Table 3, we find that trust in leaders accounts for 27.7% of the total indirect effects. The indirect effect through defensive silence accounted for 48.6% of the total indirect effect. The chained mediation effects of trust in leaders and defensive silence accounted for 23.7% of the total indirect effects. Thus, considering the individual mediation variables, defensive silence has the largest indirect effect on ICWB, followed by trust in leaders, and finally, the chained mediation effect of trust in leaders and defensive silence.

## 5. Conclusions

### 5.1. Discussion

The present study aimed to explore the impact of differentiated empowering leadership on employees’ ICWB, as well as the mediating roles of trust in leaders and defensive silence between them. The empirical results largely support our hypotheses. Specifically, trust in leaders and defensive silence are significant predictors of employees’ ICWB. However, the direct impact of differentiated empowering leadership on ICWB is not significant. Moreover, it has been observed that trust in leaders and defensive silence both serve as intermediary factors in the relationship between differentiated empowering leadership and ICWB. Additionally, a chained mediation effect is also present. In other words, differentiated empowering leadership indirectly influenced employees’ ICWB by decreasing trust in leaders and increasing defensive silence.

Our speculation that Hypothesis 1 is rejected can be attributed to the influence of cultural value orientations on Chinese employees. Organizational culture in China often exhibits a high-power distance. Power distance indicates the extent to which individuals accept uneven power distribution in social institutions or organizations. People with high power distance believe in a hierarchical structure, accepting and expecting respect for authority. As a result, in cultures with high power distance, workplace inequalities are largely rationalized. The research by Anand et al. [67] also suggests that a high power distance orientation weakens the norm of reciprocity in social exchange relationships. Additionally, “collectivism” might offer an explanation. Specifically, the operational assumption of collectivism is that groups constrain individuals and individuals have mutual obligations [68]. In this working environment, individuals are viewed as part of the collective, which encourages prioritizing collective interests over individual interests [69]. As a result, even when leaders treat team members differently, employees remain bound by organizational norms and collective-imposed obligations. This can lead to employees displaying defensive silence and other passive behavioral responses, rather than engaging in more destructive counterproductive work behaviors. This perspective is consistent with the findings of Erdogan and Liden [70], who observed that among collectivists in the Turkish textile industry, maintaining harmonious interpersonal relationships is prioritized over retaliating against fairness norm violations.

Furthermore, it is important to note that while the observed mediation effects provide valuable insights into the mechanisms linking differentiated empowering leadership and ICWB, caution is required when interpreting these results. A key issue is the reliance on statistical significance as the primary indicator of mediation [71]. As Kline [72] emphasized, statistical significance does not necessarily reflect the practical importance of an effect. In mediation analysis, the *p*-value indicates the probability of observing the data under the null hypothesis but does not convey the effect size or its practical significance. This can lead to an overemphasis on findings that may not have substantial real-world implications. Therefore, it is more important to discuss the results in the context of their practical significance, rather than focusing solely on statistical significance.

In conclusion, our study provides a simplified explanation for the complex relationships among differentiated empowering leadership, trust in leaders, defensive silence, and employees’ ICWB. These findings have both theoretical and practical implications.

### 5.2. Theoretical Implications

Currently, the mechanism by which differentiated empowering leadership influences deviant employee behavior is still being explored. Owing to variations in power distance, individualism versus collectivism, and task-oriented versus relationship-oriented, different cultural values may lead to diverse effects in this mechanism, thus warranting further validation. The core concepts of Chinese cultural values, such as collectivism, hierarchical order, “face”, and “guanxi”, might influence the degree to which Chinese employees accept differentiated empowering leadership behavior [73]. Our study not only responds to the call by Sharma and Kirkman [74] to explore “the less positive and unexpected negative consequences of empowering leadership” but also provides a new perspective for explaining the diversity and complexity of leadership behavior in cross-cultural research (Chinese context). To the best of our knowledge, this study is the first empirical study to explore the chained mediation effect of differentiated empowering leadership.

Additionally, we enrich the understanding of the antecedents and outcomes of trust in leaders and defensive silence in different cultural backgrounds. In particular, we highlight the role of defensive silence as a conscious self-protective behavior adopted by employees. Unlike previous studies that treated employee silence as a unified construct, we differentiated between passive and compliant acquiescent silence and prosocial and active prosocial silence. By focusing on employees’ defensive silence, we emphasize the self-protective strategies that employees may employ when faced with differentiated empowering leadership. This study offers novel insights into the adverse impacts of differentiated empowering leadership on employee attitudes and behaviors.

### 5.3. Practical Implications

The findings of this study offer empirical evidence to support management practices. We emphasize the negative impact of leaders’ differential empowerment behavior on employees’ deviant behavior. The intention of leadership empowerment is to encourage subordinates to make autonomous decisions, take risks, and actively contribute to work outcomes [4]. However, a higher degree of differential treatment increases team members’ perception of being “in-group” or “out-group”. When employees perceive a lack of trust and recognition in the organization, it lowers their moral judgment of leaders, damages trust in the leader, and manifests behaviors such as passive resistance, malicious gossip, and counterproductive work behavior [75]. Even “in-group” members are not immune to social comparisons [40]. Because the distinction between “in” and “out” of the circle is relative, there are also core members within the “in” circle who have more advantageous resource allocations. This upward comparison tendency leads to the majority of members perceiving themselves as being at a resource disadvantage.

Therefore, we recommend that leaders should strengthen communication with their subordinates and reduce social distance. For instance, adopting informal methods of interaction can help in promptly understanding and addressing subordinates’ emotional shifts. Providing extra emotional support to those in disadvantaged positions is crucial for timely alleviating their negative emotions, thereby reducing adverse reactions. Furthermore, organizations should offer empowerment training to leaders to enhance their delegation skills. Such training can help managers understand the necessity, timing, and methods of empowerment. Empowerment should be appropriate and moderate, with its degree and scope matching the subordinates’ abilities, experiences, and developmental needs. To achieve these objectives, organizations can invite experts to deliver lectures or conduct personalized coaching sessions, offering one-on-one mentorship to emerging leaders seeking to enhance their skills.

### 5.4. Limitations and Future Directions

The current study has certain limitations. First, it did not consider the influence of employees’ individual traits. Prior research has demonstrated a correlation between employee personality traits (such as extraversion, neuroticism, conscientiousness, and proactivity), trust in leaders, and defensive silence [76,77]. Personality traits may play a moderating role in the relationship between differentiated empowering leadership and subordinate attitudes and behaviors. Therefore, future research should consider the moderating effects of employee personality traits and explore whether the impact of differentiated empowering leadership on trust and defensive silence varies across personality traits.

Second, this study utilizes cross-sectional data and questionnaire surveys conducted over a one-month period. Future research could enhance the value of this study by employing longitudinal tracking surveys and a mixed design that combines self-reported data and a paired research design. Longitudinal tracking surveys allow researchers to observe long-term effects and trends in the relationship between leadership behaviors and employee attitudes and behaviors. Self-reported data can be collected at multiple timepoints. Paired data can match leaders with their subordinates to compare the impact of differentiated empowering leadership on trust and defensive silence among different employees, thereby gaining a better understanding of the influence of individual differences and supervisor–subordinate interactions on outcomes.

Although this study provides valuable insights into the mediating role of trust in leaders and defensive silence in the relationship between differentiated empowering leadership and ICWB, it is important to recognize that other theoretical perspectives might also be relevant. For example, psychological contract theory offers an alternative perspective. This theory suggests that there is an implicit agreement of mutual beliefs, expectations, and obligations between employers and employees. When empowerment is distributed unequally, employees may feel that the organization has violated this contract, leading to negative emotions such as anger and betrayal. These emotions can then result in defensive silence and counterproductive behaviors. Future research should explore integrating these alternative theoretical perspectives to provide a more comprehensive understanding of how differentiated empowering leadership impacts employee behavior. This could help in identifying new mechanisms and strategies to mitigate the potential negative effects of such leadership, further enriching both the theoretical and practical contributions in this field.

## Figures and Tables

**Table 1:** Descriptive statistics and correlations among the study variables.

Variable	Mean	SD	1	2	3	4	5	6	7	8
1 Gender	1.442	0.497	NA							
2 Age	2.873	1.469	0.023	NA						
3 Edu	2.805	0.892	−0.026	−0.044	NA					
4 Tenure	2.247	1.404	−0.021	0.834 **	−0.105	NA				
5 DEL	0.144	0.131	0.002	0.070	0.141 *	0.051	(0.941)			
6 TL	3.803	0.682	0.010	0.026	−0.068	0.076	−0.324 **	(0.910)		
7 DS	2.496	0.860	−0.020	−0.030	0.049	−0.041	0.291 **	−0.362 **	(0.883)	
8 ICWB	2.285	0.925	−0.076	−0.259 **	−0.149 **	−0.258 **	−0.020	−0.229 **	0.391 **	(0.920)

Note(s): *n* = 308. DEL represents differentiated empowering leadership, TL represents trust in leaders, DS represents defensive silence, ICWB represents interpersonal counterproductive work behaviors; internal consistency reliability (α) coefficients are reported in parentheses, and NA = not applicable. * *p* < 0.05, ** *p* < 0.01.

**Table 2:** Results of regression analysis.

Variable	TL	DS		ICWB			
	Model 1	Model 2	Model 3	Model 4	Model 5	Model 6	Model 7
Gender	0.016	−0.022	−0.017	−0.082	−0.078	−0.073	−0.072
Age	−0.095	−0.012	−0.040	−0.110	−0.133	−0.105	−0.118
Edu	−0.008	0.002	0.000	−0.179 **	−0.180 **	−0.179 ***	−0.180 ***
Tenure	0.171	−0.046	0.005	−0.188	−0.146	−0.168	−0.148
DEL	−0.325 ***	0.294 ***	0.197 **	0.022	−0.057	−0.100	−0.132 *
TL			−0.297 ***		−0.244 ***		−0.132 *
DS						0.417 ***	0.380 ***
R^2^	0.116	0.088	0.166	0.109	0.162	0.268	0.282
△F	35.096 ***	27.856 ***	28.167 ***	0.163	18.949 ***	65.353 ***	50.202 ***

Note(s): *n* = 308. * *p* < 0.05, ** *p* < 0.01, *** *p* < 0.001.

**Table 3:** Estimation and test results of the mediating effects.

Hypothesis	Effect	Boot SE	Boot LLCI	Boot ULCI	Proportion
Total indirect effect	1.091	0.219	0.693	1.535	-
H2: DEL → TL → ICWB	0.302	0.146	0.034	0.614	27.7%
H3: DEL → DS → ICWB	0.530	0.181	0.202	0.912	48.6%
H4: DEL → TL → DS → ICWB	0.259	0.082	0.118	0.435	23.7%

Note(s): *n* = 308.

## Data Availability

The data supporting this study’s findings are available from the corresponding author upon reasonable request.
